# Conservation of *Apis mellifera mellifera* L. in the Middle Ural: A Review of Genetic Diversity, Ecological Adaptation, and Breeding Perspectives

**DOI:** 10.3390/insects16050512

**Published:** 2025-05-11

**Authors:** Olga Frunze, Alexander V. Petukhov, Anna Z. Brandorf, Mikhail K. Simankov, Hyunjee Kim, Hyung-Wook Kwon

**Affiliations:** 1 Department of Life Sciences & Convergence Research Center for Insect Vectors (CRCIV), Incheon National University, R&D Complex, Incheon 22012, Republic of Korea; frunzeon@gmail.com (O.F.);; 2 Department of Ecology, Perm State Agro-Technological University named D.N. Pryanishnikov, Perm 614990, Russia; 3 Federal Agricultural Research Center of the North-East named N. V. Rudnitskogo, Russian Academy of Science, Kirov 610007, Russia

**Keywords:** *A. m. mellifera* L., local honey bees, Prikamskaya population, bee breeding, queen rearing, ecology, physiology

## Abstract

The European dark bee is well adapted to cold winters and short summers. However, threats from habitat loss, pests, and hybridization with southern bees pose significant challenges to its populations. The Perm region (Middle Ural, Russia) hosts a distinct population of *Apis mellifera mellifera*, known as Prikamskaya. Despite extensive local research, a comprehensive analysis remains lacking. This review presents an analysis based on selected historical, ecological, genetic, and regulatory sources relevant to honey bee populations in northern climates. Inclusion criteria prioritized peer-reviewed scientific literature, regional monographs, institutional reports, and expert contributions published from the 20th century onward. Preference was given to studies addressing environmental conditions, queen-rearing practices, population structure, and conservation strategies. At the northern limit of honey bee distribution, the region has diverse forest zones and a growing season of 145–190 days, influencing nectar availability from lime, honeysuckle, and willow. Although the region’s potential honey yield is estimated at 390,919,300 kg, only 6.7% of its 3,007,200 colonies are commercially utilized, largely due to the low number of apiaries specializing in local honey bees. Distinct northern and southern types of *A. m. mellifera* have been identified based on morphological (cubital index) and physiological (cold resistance) traits, although links to genetic diversity remain underexplored. This study underscores the importance of regional conservation efforts in preserving the genetic diversity of *A. m. mellifera*, emphasizing the need for targeted breeding strategies to address climate change and hybridization, ensuring the sustainability of agriculture and natural ecosystems worldwide

## Simple Summary

The European dark bee (*Apis mellifera mellifera*) is native to northern, central, and western Europe and has adapted to cold winters and short summers. However, habitat loss, pests, and hybridization with southern bee breeds threaten its populations, making conservation a priority. In the Middle Ural (Perm region, Russia), a distinct *A. m. mellifera* population, Prikamskaya, has persisted since the 12th century. This review presents the ecological conditions, population dynamics, and genetic differentiation of this subspecies, focusing on its adaptation to harsh northern climates. The region, located at the northern limit of honey bee distribution, features diverse forest zones and a short growing season, which shape floral resource availability. Although genetic analyses suggest distinctions between north and southern bee types, the current literature provides limited direct evidence linking genetic diversity to specific morphological traits (e.g., cubital index) or physiological adaptations (e.g., cold resistance). Despite an estimated honey yield of nearly 400,000 tons, only a small fraction of colonies are commercially utilized, highlighting the underuse of this region’s beekeeping potential. Our findings emphasize the need for conservation strategies to maintain the genetic integrity of *A. m. mellifera*, ensuring its role in pollination, ecosystem stability, and climate resilience.

## 1. Introduction

*Species Apis mellifera* L. honey bees play a pivotal role in agriculture by pollinating a wide variety of fruits and vegetables and obtaining seeds, as well as producing honey, propolis, royal jelly, and other valuable substances used for human food and medicine. Among the 33 subspecies of *A. mellifera* [1], *A. m. mellifera* Linnaeus 1785 (European dark bee, black bees, Central Russian bee) is well adapted to cold winters and short summers [2], which is why it has historically been bred in Europe [3] and Russia [4] . This subspecies has evolved traits such as efficient winter clustering and conservative brood rearing to survive harsh climates [3]. However, its populations have declined due to habitat loss [5], climate change [6], hybridization with southern bee breeds, and modern beekeeping practices that favor other *A. mellifera* subspecies [7,8] . Moreover, winter mortality in *A. mellifera* remains a crucial issue for apiaries [9,10], and honey bee conservation has become a major global concern.

To address these threats, SICAMM (the International Association for the Protection of the European Dark Bee) was established in 1994 to support the conservation, study, and promotion of *A. m. mellifera*. The organization connects beekeepers, researchers, and conservationists across Europe, facilitating collaboration to protect this native honey bee subspecies and ensure its long-term survival [11]. Another network, COLOSS (Prevention of Honey Bee COlony LOSSes), has been an international association since 2008, whose members have published comprehensive, integrated survey results to understand the causes of honey bee colony losses and to identify strategies for mitigating contributing factors, including those affecting *A. m. mellifera* [12].

Building on global research efforts, such as those by SICAMM and COLOSS, understanding honey bee colony losses is also crucial in the context of the Russian Federation (later Russia), where *A. m. mellifera* plays a significant role in the beekeeping industry. For this, the Breeding Center for the Central Russian Honey Bee (Russian Association for Conservation of *Apis mellifera mellifera* L., RACAMM) was founded in December 2012 based on the North–East Regional Agrarian Scientific Center (https://apis-mellifera-mellifera-l.ru/en/ accessed on 1 February 2025). Their aim is the implementation of scientific and methodological services and information support of breeding work with black bees (*Apis mellifera mellifera* L.) in Russia.

In the last century, beekeepers primarily bred *A. m. mellifera* L. (60%) among the total number of honey bee colonies, which reached 10 million. In modern beekeeping, as a result of mass introduction, there are currently three million bee colonies in Russia, with *A. m. mellifera* L. making up 4% [13]. To save the honey bee population of *A. m. mellifera*, which is particularly important due to the declining trend, certified breeding centers for the reproduction of *A. m. mellifera* currently operate in the Altai and Perm regions, as well as in the Republics of Bashkortostan, Tatarstan, and Udmurtia. Additionally, there are three state-certified centers and parks dedicated to the breeding of *A. m. mellifera*: Shulgan-Tash State Nature Reserve (Republic of Bashkortostan, https://shulgan-tash.ru/ accessed on 1 February 2025), Vishersky wildlife sanctuary, and Malinovii Hutor reserve (Perm region, https://www.vishersky.ru/ru/organizaciya/dokumenty accessed on 1 February 2025 and https://uinsk.ru/ accessed on 1 February 2025, respectively).

Within the subspecies *A. m. mellifera*, which inhabit vast areas, distinct populations have been identified, selectively bred, and officially certified in Russia. These include the Bashkirsky (recognized as a new breed), as well as regional breed types and populations such as Priokskaya, Orlovskaya, Tatarskaya, Burzyanskaya Bortevaya, and Prikamskaya [14]. Since an overview of beekeeping in Russia has already been published [15], this review specifically focuses on local apiculture in the Perm region, because it is (160,000 km^2^) larger than countries, such as Portugal (92,000 km^2^), Hungary (93,000 km^2^), Greece (132,000 km^2^), and South Korea (100,000 km^2^). Moreover, the northern limit of the range of *Apis mellifera* L. occurred at the Perm region at around 58.0130° N (Krasnovishersk city). This vast area may naturally isolate honey bees for local breeding; moreover, this local population’s importance lies in their unique adaptations to regional environmental conditions, which are essential for resilience and sustainability in honey bees [16]. The Perm region’s extreme northern climate and long-standing beekeeping tradition, dating back to the 12th century, offers a unique setting for studying the genetic, behavioral, and environmental adaptations of honey bees, yet a comprehensive review of the existing research has not yet been conducted.

A broad spectrum of sources was reviewed, including peer-reviewed scientific articles, archival documents, academic theses, and monographs, with a particular focus on works from the late 1970s onward. Key contributions by Professor Arkadiy Ivanovich Shurakov, Ph.D., and Academician Professor Evgeny Konstantinovich Eskov, who helped identify *A. m. mellifera* in the region, were foundational to the subsequent research used in this analysis. Additional research from the Department of Zoology at Perm State Pedagogical University and Perm State Agricultural Academy, led by Assistant Professors Alexander Vasilyevich Petukhov, Ph.D., and Mikhail Kimovich Simankov, Ph.D., provided essential insights into local conservation and breeding practices, as well as honey bee certification.

To capture both historical and contemporary perspectives, a conceptual database was compiled that included foundational morphometric studies (e.g., [4]) and recent genetic and management research. Russian-language materials formed the bulk of the evidence, reflecting the regional academic tradition and limiting access through English-only search platforms. This multilingual approach allowed for a more comprehensive understanding of local apicultural development. Additional data were collected from online resources [17] and local experts, ensuring both depth and regional accuracy in the analysis.

The current review explores how the specific environmental pressures of the Perm region shape the characteristics of local honey bees. Our key objectives were to (1) present the environmental conditions, food sources, and honey bee wild reserve in Perm region; (2) describe the *A. m. mellifera* honey bees Prikamskaya population; (3) discover the features of queen rearing in northern climate; and (4) investigate state regulation in beekeeping and genetic conservation. By synthesizing research spanning several decades, we provide a long-term perspective on the characteristics of honey bee populations and their adaptation strategies in response to environmental and anthropogenic pressures. This study targeted conservation efforts to maintain genetic diversity and ecological stability within *A. m. mellifera* populations.

## 2. Ecological Conditions for Beekeeping in Perm Region

The Perm region, located in the European part of Russia at the geographical intersection of Europe and Asia, stretches along the western slopes of the Middle and Northern Ural Mountains. Its coordinates range from 61°39′ N to 56°06′ N latitude and 60°35′ E to 60°50′ E longitude [18]. Spanning approximately 645 km from north to south and 420 km from west to east, it marks the northern boundary of honey bee distribution, around 58.0130° N (Krasnovishersk).

Long-term climatic conditions play a critical role in shaping the physiology, behavior, and survival of honey bee populations, particularly in regions with extreme environmental variability. For this review, we examined monthly baseline temperature data for the Perm region spanning the period 1948–2020, using a publicly accessible historical weather records database (https://weatherspark.com/h/y/148901/1960/Historical-Weather-during-1960-at-Perm-International-Airport-Russia accessed on 1 February 2025). This temporal dataset allowed us to explore trends in seasonal temperature fluctuations and the potential ecological pressures they exert on *A. m. mellifera* colonies in a region characterized by long, cold winters and short summers.

### 2.1. Climate Trends and Regional Temperature Variability

Because honey bee flight activity is largely determined by temperature, researchers have long been interested in their cold resistance [19]. Their foraging efficiency peaks between 20 and 30 °C, within a broader range of 10–40 °C [20,21]. However, recent climate change trends are disrupting insect developmental rhythms and affecting nectar availability [22], with temperature playing a key role. Notably, honey bees and nectar-secreting plants exhibit similar temperature sensitivities. Plants do not secrete nectar optimally when temperatures fall below 10 °C or rise above 35–38 °C, with the ideal range being 16–26 °C [23]. Given these interconnections, detecting climate change trends is crucial for understanding honey bee adaptation and forecasting both their survival in the wild and the success of beekeeping in affected regions.

The annual temperature trend was analyzed only in the capital city (Bolshoe Savino Airport, Perm), as this location has the oldest freely available weather records in the Perm region. No significant differences were found in the average temperatures over the past 74 years. However, projections indicate that by 2025, the average temperature will be nearly 3 °C higher than in the 1950s–1970s [24], even if this increase is not statistically significant in monthly analyses. Public reports on snow cover duration were considered to further assess climate change trends. Historically, snow cover lasted for 190 days per year (more than six months), but after 70 years, it now persists for only 167 days (5.6 months) [24]. These findings indicate that climate change is gradually affecting the Perm region, leading to shorter winter conditions. While this may not initially seem problematic, sudden fluctuations in freezing temperatures (ranging from 0 °C to −15 °C or lower), especially towards the end of overwintering, could threaten colony survival.

**Figure 1:**
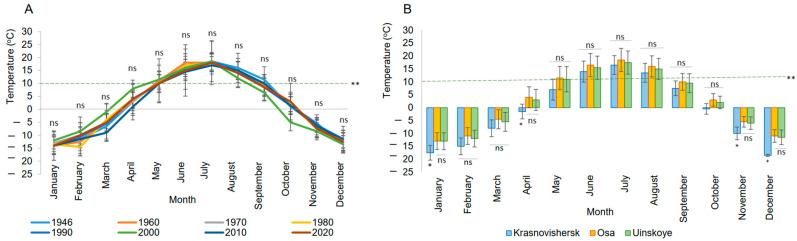
Temperature trends and regional variations in the Perm region. (**A**) Annual temperature trend in the capital city (Bolshoe Savino airport, Perm city) over 74 years as a climate change reference. (**B**) Comparison of monthly temperatures between the northern (Krasnovishersk, city) and southern (Osa, city; Uinskoye, city) breeding centers in the Perm region (2024). ns—not significant; *—significant differences, one-way ANOVA, Duncan post-hoc test, *p* ˂ 0.05; ** indicates temperatures below the threshold at which nectar secretion decreases and honey bee activity ceases.

A key characteristic of *A. m. mellifera* in the Prikamskaya population is its adaptation to a prolonged overwintering period. The duration of overwintering (winter diapause) is temperature-dependent, as individual honey bees cannot fly or physiologically respond to ambient temperatures below 10 °C [21] or 12 °C [20]. In the southern Perm region, temperatures remain below 10 °C for seven months, from October to April (Figure 1A), during which honey bees survive in winter diapause by forming a thermoregulating cluster [25]. In northern areas (Krasnovishersk), overwintering extends to eight months, beginning in September, due to lower temperatures (Figure 1B). Significant temperature differences were detected between the northern and southern Perm areas, with the north (Krasnovishersk) experiencing notably colder conditions from November to January and in April (Figure 1B). Consequently, wild honey bees in the north require more energy to survive. As climate change alters seasonal temperature patterns, honey bee populations may need to adjust their overwintering behavior, potentially affecting their survival rates.

Another key characteristic of *A. m. mellifera* in the Prikamskaya population is its adaptation to a short honey collection period. Although plant growth lasts for five months when temperatures are above 10 °C (Figure 1A,B), rain and wind also have a depressive influence on honey bee activity and the cessation of blooming for nectar collection. While nectar flow can begin as early as May in some years in the southern areas, it predominantly occurs in July. Beekeeping records from 2014 to 2019 (southern Perm region, Osa city, southern Prikamskaya population, Osinskaya) show considerable variation in the duration of the main honey flow, ranging from just five days in early August (2014) to 30 days in July (2016), with other years falling in between: eight days from late July to early August (2017), 19 days in July (2018), and 10 days at the end of July (2019) [26]. The main honey flow refers to the time when honey bees intensively gather nectar, which beekeepers then harvest for commercial sale. On other days, foraging activity was limited due to rain or cold temperatures, which reduced nectar secretion. These variations highlight the influence of weather conditions on nectar flow and the potential impact of climate change on honey flow.

To conclude, while the shortening of overwintering and the warmth of summer may not pose a significant issue, sudden temperature fluctuations can jeopardize colony survival. Monitoring these changes is crucial for understanding their impact on honey bee health, agriculture, and beekeeping success.

### 2.2. Forest Zones and Nectar Resources

**Figure 2:**
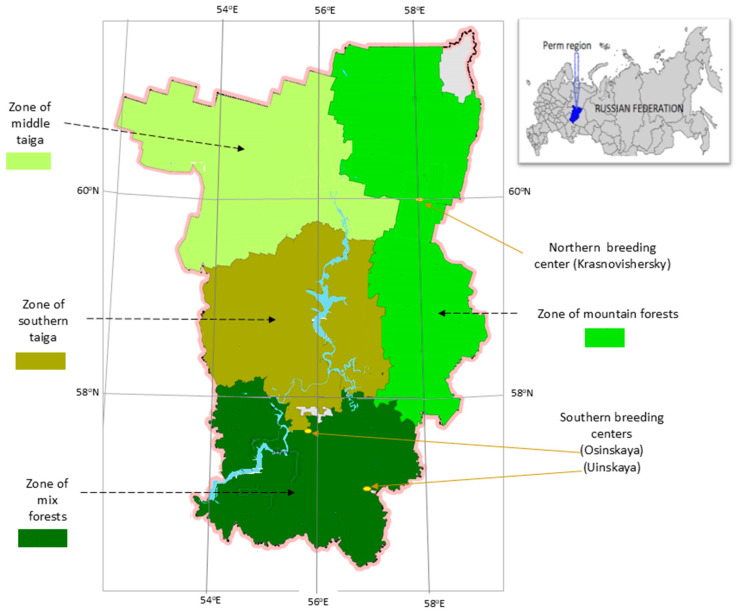
Ecological zoning and the northern and southern breeding centers of *A. m. mellifera* L. honey bees (Prikamskaya population) in the Perm region.

Temperature changes strongly influence the abundance of melliferous plants, which are part of ecosystems across various global climate zones [27]. The boreal forest zones, with some areas of mixed forest, belong to the larger Siberian taiga but are located further west, covering more than half of the Perm region. This region is classified into four distinct ecological–geographical zones: mountain forests, middle taiga, southern taiga, and mixed forests (Figure 2). Vegetation composition varies across these zones, influencing the length of the growing season, which lasts approximately 145 days in the northern part, 165 days in the central part, and 190 days in the southern part [28]. These variations affect honey bee habitats, the diversity of melliferous plants, and nectar availability. For instance, in the southern taiga, tree species such as lindens, elms, and maples are abundant, whereas these species are absent in the middle taiga and farther north [29]. The main nectar source is primarily concentrated on linden (261,100 hectares), with additional sources in the southern taiga, including meadowsweet (100,700 hectares), goutweed (164,900 hectares), hogweed (178,950 hectares), and mullein, while in the middle taiga, it is complemented by meadowsweet, goutweed, and heather [30].

The region’s nectar resources are dominated by melliferous plants, with lime trees contributing approximately 509,119.5 kg of honey per growing season. Other major nectar sources include honeysuckle (55,421.9 kg), willow (41,468.0 kg), rowan (36,304.7 kg), rose hip (13,194.7 kg), and goutweed (58,493.3 kg) [29].

Calculations were based on weather conditions, nectar secretion rates of key melliferous plants, flowering duration, and plant cover area to estimate honey yield across forested areas. The estimated yields per forest zone were as follows: 143,425,2000 kg from the mixed forest zone, 137,465,100 kg from the southern taiga, 74,705,000 kg from the mountain forest zone, and 35,324,000 kg from the middle taiga [29], with a total estimated honey yield of 390,919,300 kg.

However, a cost–benefit analysis revealed that only 6.7% of the region’s 3,007,200 honey bee colonies are commercially utilized [29], underscoring the underdevelopment of the apiculture industry and its unrealized economic potential.

### 2.3. Bee-Breeding Centers and Reserve for A. m. mellifera Honey Bees Prikamskaya Population

Despite the general underdevelopment of apiculture concerning the abundance of melliferous plants, both private and commercial beekeeping are practiced in the Perm region. Beekeeping centers have been established in both the northern (Krasnovishersky) and southern (Osinskaya) parts of the region, approximately 359 km apart. Additionally, two southern centers, Osinskaya and Uinskaya, are located 81 km apart (Figure 2).

Northern (Krasnovishersky) Prikamskaya population of *A. m. mellifera* L. honey bees were bred in apiaries in the northern part of the Perm region and sold by the company “Perm Bees” (https://perm-bees.ru accessed on 1 February 2025). The last company operates three apiaries in the region: one stationary with 100 colonies, one migratory with 50 colonies for transport to lime blossom locations, and one breeding apiary with 50 northern colonies.

Since 1998, the former honey-bee-breeding station ‘Nizhniy Sypovskoi’ (Uinsky municipal district, Perm region) has preserved 1090 colonies to protect the southern (Uinskaya) Prikamskaya population of *A. m. mellifera* L. (https://uinsk.ru/sotsialnaya-sfera/spisok-selskohozyajstvennyh-predpriyatij-rajona/ accessed on 1 February 2025). Similarly, in 1991, the company “Tentorium” established the “Parasol” breeding station with 645 colonies to conserve the same population (https://tentorium.ru/parasol/ accessed on 1 February 2025).

**Figure 3:**
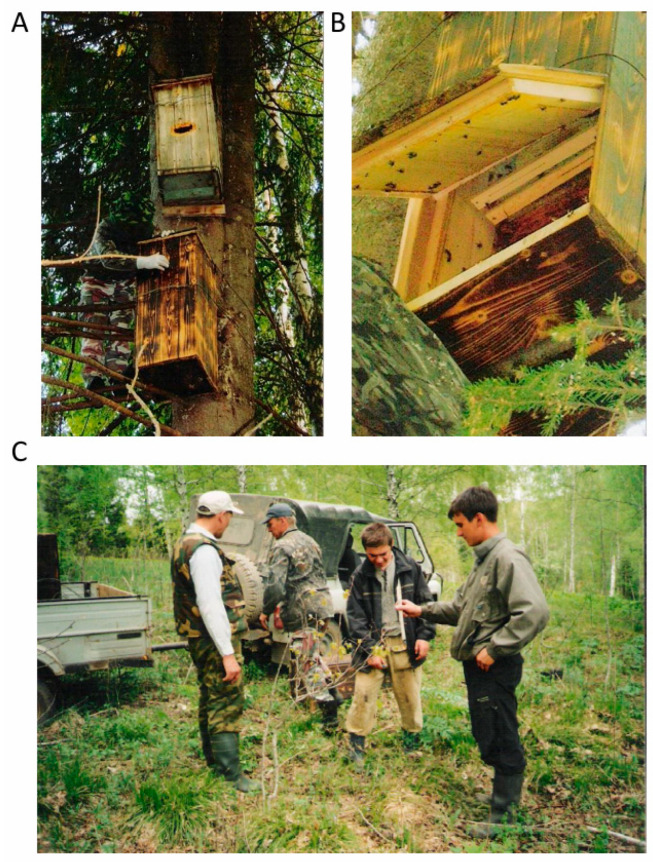
Installation of artificial dwellings for wild *A. m. mellifera* L. honey bees in the “Malinovyi Hutor” reserve, Perm region. (**A**,**B**)—Artificial dwellings for honey bees; (**C**)—Alexander V. Petukhov (left) and honey bee researchers at the “Malinovyi Hutor” reserve.

One of the key achievements of the research group was the establishment of the first Perm Reserve, “Malinovyi Hutor,” in 2002, aimed at protecting southern (Uinskaya) honey bees and preserving the natural gene pool of *A. m. mellifera* L. (Prikamskaya population) [31]. The reserve covers an area of approximately 50 km^2^ and includes 84 species of honey plants, providing a rich nectar base for honey bee colonies. Additionally, artificial dwellings were placed to increase the local honey bee population (Figure 3). This supports colony development during the early spring and spring–summer periods [32].

In conclusion, both private and commercial beekeeping contribute to the preservation of the Prikamskaya population in the Perm region. Efforts such as breeding stations and the establishment of the “Malinovyi Hutor” reserve have played some role in conserving *A. m. mellifera* L. and maintaining their genetic integrity. These efforts also have the potential for further growth, both as a wildlife park and in the development of commercial beekeeping apiaries.

## 3. Characteristics of *A. m. mellifera* L. Honey Bees (Prikamskaya Population)

The *A. m. mellifera* L. honey bee breed type has been developed over an extended period in the conditions of the Perm region. The primary aim of honey bee studies was to assess local honey bee populations based on ecological, morphological, and genetic characteristics, ensuring their conservation in the natural environment.

### 3.1. Ecologic Characteristics

Ecological characteristics refer to organisms’ adaptive traits and behaviors that define their interactions with the environment [33]. In this review, these characteristics were assessed for the Prikamskaya population as a whole, without differentiating between northern and southern groups.

A significant ecological adaptation of the *A. m. mellifera* Prikamskaya population is its ability to successfully overwinter for up to six months in southern regions (from November) and up to seven months in northern regions (from October to April). For this, *A. m. mellifera* maintains a carbon dioxide concentration within the typical range of around 5% [34], whereas the overall range for honey bees varies from 4% to 8% [35]. These carbon dioxide levels at relatively low temperatures lead to reduced food consumption, as their combined effects intensify the suppression of bee activity [36].

Another important ecological trait of *A. m. mellifera* honey bees was their rapid colony development during the warm season, when the egg-laying capacity of the queen reached 2043–2460 eggs per day [37,38,39]. For the Prikamskaya population, this value was 1928 ± 289 eggs per day at the end of June for a first-year queen [40]. By August, queen egg-laying activity decreases, leading to a lack of brood by September [41]. Furthermore, the presence of brood in honey bee colonies as late as October may indicate hybridization with southern bee lineages. In southern regions, colony growth lasts for approximately six months, from May to October, whereas in northern regions, this period is slightly shorter, lasting about five months until September. This is balanced by an extended broodless period during winter diapause.

Another notable ecological feature is that honey bees have a short swarm period [42,43] and efficiently utilize the period of abundant, short honey flows, with the highest productivity occurring in July in the Perm region [44]. This productivity is reflected in the nectar crop mass of foragers, which determines the daily nectar income. For example, the mass of the nectar crop of foraging *A. m. mellifera* honey bees (population not reported) was 49.20 ± 3.48 mg [45], while foraging bees of the *A. m. mellifera* Prikamskaya population gained 72.20 ± 1.15 mg/bee [46], indicating superior foraging efficiency. When the daily nectar intake of *A. mellifera* honey bee colonies in the Pskov region reached a maximum of 2 kg/day [47], in *A. m. mellifera* from Bashkiria (Bashkirskaya population), it was 2.7 kg/day [48]. For the Prikamskaya population, the nectar intake per standard colony with 2.5 kg of workers (approximately 25,000 bees) reached 4.5 kg/day [26], resulting in a total honey yield of 64.0 ± 1.50 kg per colony during a high honey flow [46].

The Prikamskaya population of honey bees demonstrates a high level of resistance to nosemosis and honeydew toxicosis, consistent with other *A. m. mellifera* populations [49]. In the spring of 2013, nosemosis prevalence was assessed in bee samples collected from six apiaries in the Perm region (Perm, Uinsky, and Kungursky districts), encompassing a total of 384 colonies. Of the 56 colonies analyzed, 41 were identified as the native *A. m. mellifera*, while 15 belonged to the introduced *A. m. carpathica* [50]. The introduced bees exhibited a higher incidence of infection, with 12 out of 15 colonies (80%) affected and infestation levels ranging from 70% to 100%. In contrast, native Central Russian bees displayed significantly lower infection rates, with contamination levels ranging from 0% to 20% [51].

However, the mechanism of *A. m. melllifera* resistance to *Varroa* mites [44] remains a subject of ongoing debate. A shorter brood development period during summer appears to reduce *Varroa* mite infestation time, but it is not resistant. Resistance of *A. m. mellifera* in Ireland was reported as a specific behavior that has been selectively bred against *Varroa* [52]. So, there is a need to research more for breeding honey bees with *Varroa*-resistant behavior.

In conclusion, the *A. m. mellifera* Prikamskaya population exhibits several notable ecological adaptations that contribute to its resilience and productivity. These include efficient overwintering strategies, rapid colony development during the warm season, and high foraging efficiency, especially during short honey flows. The population also demonstrates resistance to diseases like nosemosis and honeydew toxicosis. However, the issue of *Varroa* mite resistance remains unresolved and requires further investigation. Overall, these ecological traits highlight the adaptability and strength of the Prikamskaya honey bee population, underscoring the need for continued research to enhance its resilience to emerging challenges.

### 3.2. Physiological Adaptations During Overwintering

Physiological adaptations are the internal biochemical processes that enable organisms to survive in specific environmental conditions [53]. Physiological studies examined the Prikamskaya population as a whole, without differentiating between northern and southern groups.

One key area of investigation has been the water content in overwintered honey bees and their temperature at maximum hypothermia [32,54]. It was found that honey bees with lower water content had a lower maximum hypothermia threshold, indicating greater cold resistance. Notably, the native *A. m. mellifera* L. exhibited consistently lower water content compared to the introduced *A. m. carpatica*, suggesting an adaptation of the former to prolonged overwintering conditions [32].

Histological studies have provided further insights into the secretory activity of the intestinal epithelium, an important factor influencing winter survival [55,56]. Increased secretory activity was observed at the onset of overwintering, implying additional energy expenditure for nest thermoregulation [57]. This phenomenon reflects the physiological adjustments required for successful overwintering.

Enzyme activity, particularly in the oxidative stress response, has been another area of focus [58,59,60]. Catalase activity, which plays a crucial role in neutralizing reactive oxygen species, was shown to decline by January before increasing toward the end of overwintering [59]. Additionally, phosphatase activity, which reflects energy demands, varied depending on a bee’s position within the winter cluster, emphasizing the role of thermoregulation in colony survival [61]. At that time, studies showed that introduced *A. m. carpatica* honey bees exhibited higher catalase and phosphatase activity compared to native *A. m. mellifera*, suggesting that the introduced bees initiated metabolic activity and brood rearing earlier than the native population [58]. This fluctuation was linked to metabolic suppression during the deep overwintering phase and the subsequent reactivation of metabolic processes in preparation for brood rearing.

To conclude, the physiology of overwintering *A. m. mellifera* L. honey bees was studied in comparison with the introduced *A. m. carpathica*. Low water contents, increased secretory activity of the intestinal epithelium, and prolonged metabolic suppression suggest a genetically programmed long overwintering period for the *A. m. mellifera* Prikamskaya population of honey bees.

### 3.3. Morphometric Characteristics of Northern and Southern Prikamskaya Population of Honey Bees

Morphological characteristics refer to the structural and anatomical features of an organism, which are influenced by genetic and environmental factors and play a role in adaptation and survival. The measured characteristics derived from them are referred to as morphometric and are well described for honey bees [62,63].

Morphometric identification of the northern (Krasnovisherskaya) and the southern (Osinskaya, Uinskaya) Prikamskaya population was carried out based on eight features, such as the length of the proboscis, length and width of the wing, length and width of tergite 4, length of sternite 4, as well as the cubital and tarsal indexes (Table 1). All honey bees in the Perm region were compared with the standard morphometric characteristics of *A. m. mellifera* L. honey bees in Russia.

The length of the proboscis of northern and southern honey bees was minimal and within the standard range for *A. m. mellifera* L. honey bees (5.9–6.4 mm). Similarly, the average width of the forewing (3.10–3.20 mm) was within the standard range (2.9–3.3 mm) of this subspecies. Additionally, the length of the forewing in northern honey bees (9.30 ± 0.008 mm) did not exceed the standard limit for *A. m. mellifera* L., while the average length of southern honey bees was lower (9.20 ± 0.008 mm) than the standard (9.3–9.6 mm). The cubital index of southern honey bees (64.1 ± 0.65%) and northern honey bees (61.6 ± 0.34%) corresponded to the *A. m. mellifera* L. standard (60–65%). The variation in the cubital index ranged from 10.2% to 14.9%.

A morphometric identification of honey bees from 36 randomly selected apiaries (with 10 colonies each and 30 bees per colony) located in different parts of the Perm region revealed that 7.3% were pure *A. m. mellifera* L. honey bees, differing in one or two of eight standard features. Additionally, 58.5% were hybrid honey bees, and 29.3% belonged to other honey bee subspecies [29].

### 3.4. Genetic Characteristics of Northern and Southern Prikamskaya Population of Honey Bees

In-depth research was conducted on the genetic diversity of the Prikamskaya population to evaluate its purity and variability across different regions.

In collaboration with researchers from the Ufa Scientific Center of the Russian Academy of Sciences, genetic analyses of the Prikamskaya population were conducted to determine the purity of mitochondrial DNA (mtDNA) [68]. This study identified that samples of honey bees from 92 apiaries in the Perm region belong to *A. m. mellifera* subspecies, and two distinct local groups of Prikamskaya populations were identified—Northern (Krasnovishersky) and Southern (Osinskaya and Uinskaya)—based on analyses of the mitochondrial DNA COI–COII locus and two nuclear microsatellite loci (ap243 and 4a110) [66,69].

These findings provide a clearer understanding of the distinct genetic subgroups within the Prikamskaya population, offering important insights into local adaptation and conservation strategies.

## 4. Challenges and Advances in Queen Rearing of *A. m. mellifera* in the Perm Region

Queen production is a key aspect of beekeeping for maintaining honey bee colonies. The issue of queen availability has arisen due to the widespread importation of mated queens from non-native southern subspecies of honey bees [70]. This challenge is also relevant in the Perm region, where some beekeepers introduce queens from southern Russia, leading to the hybridization of native *A. m. mellifera* bees with non-native subspecies [67]. In contrast, other beekeepers working with native honey bees often prefer to rear their queens, obtained naturally during the swarming season or through supersedure, to replace aging queens and expand their apiaries [71]. However, commercial *A. m. mellifera* queen rearing is developing in the region, with specialized honey bee breeding centers such as Parasol, Perm Bees, and Nizhniy Sypovskoi focusing on the *A. m. mellifera* Prikamskaya population.

One of the main challenges in queen rearing is the short duration of warm weather, which is frequently accompanied by unfavorable conditions such as rain. In the Perm region, warm weather typically begins in late May, with the first mated queens available by June. Additionally, colonies of Central Russian bees produce a relatively small number of queen cells, ranging from a minimum of 6 to a rare maximum of 26, which further limits large-scale queen rearing [71].

Management of bred honey bee colonies begins in the preceding autumn and continues through winter and spring. Each colony is evaluated based on several criteria, including the number of overwintered bees, the absence of diseases, late-season honey productivity, and morphometric and genetic conformity to the standard for *A. m. mellifera* L. (Prikamskaya population) in the Perm region. These evaluations are conducted either individually or in commercial apiaries by professional beekeepers, who maintain the colonies in the apiary. For breeding purposes, at least five of the best colonies are selected to establish purebred lines and obtain initial queen larvae. To produce purebred mated queens, these colonies, along with those designated for drone production and nucleus colonies, are typically relocated to the mating station (Makarov, Perm Bees, personal communication).

The first step in the process is the production of virgin *A. m. mellifera* queens, which can either be sent to the customer or proceed to the next stage at the mating station. The following does not provide an overview of the general queen-rearing technique but instead highlights specific modified tips.

Several strategies for rearing virgin *A. m. mellifera* queens were tested, but since 2020, a new artificial queen-rearing method has been developed. Instead of using grafting frames, plastic queen cell cups with a wedge-shaped holder were applied. These cups are individually attached to the comb area, preferably around the brood. For queen rearing, 20–30 larvae are individually grafted into the cups and placed in the nurse colony, with an average of 15.3 ± 0.46 larvae being accepted by the bees [72]. The capped queen cells that are removed are placed in plastic cages, which are filled with 10–12 immobilized worker bees. Worker bees are immobilized by cooling them in a refrigerator or with ice to a temperature of 5–7 °C until they enter cold-induced torpor. Immobilization is necessary because filling a single cage with moved workers can be time-consuming. Additionally, immobilized bees do not sting the beekeeper and do not exhibit aggression toward the queens or other bees. This is especially crucial when working with Central Russian bees, which are known for their heightened excitability and defensiveness. After emergence, each queen is weighed and marked. The main morphological characteristics of the virgin *A. m. mellifera* queens from the Prikamskaya population, obtained by this method, are as follows: mass (209.6 ± 1.91 mg), forewing length (9.70 ± 0.039 mm), forewing width (3.28 ± 0.023 mm), length of the third tergite (3.42 ± 0.026 mm), width of the third tergite (5.71 ± 0.026 mm), and cubital index (49.6 ± 1.53%).

The next step is the production of mated queens. For that, virgin queens need to proceed with natural mating in the mating station with specially selected drones from colonies with outstanding characteristics. The *A. m. mellifera* L. honey-bee-mating station was established in an isolated area in northern Perm, in Krasnovishersk (Makarov, Perm Bees, personal communication), where the survival of wild honey bees is low. To ensure genetic isolation from wild drones, several nucleus colonies with virgin queens are tested before introducing managed drone-producing colonies. If the queens fail to mate, the area is considered suitable for use as a controlled mating apiary (personal communication).

The adaptation of the standard method for rearing and selecting *A. mellifera* queens [73] to northern climates has made it possible to produce 500–1000 mated queens per year, based on weather conditions (Makarov, Perm Bees, personal communication). However, despite these and other beekeepers’ efforts, the production of local honey bee queens still falls short of meeting the growing demand, highlighting the ongoing challenge in the industry [71]. This gap emphasizes the need for enhanced production strategies to satisfy market requirements.

## 5. Regulation in Beekeeping and Genetic Conservation: Challenges and Strategies in the Perm Region

To prevent hybridization due to the presence of various native honey bee subspecies across Russia, the Plan for Honey Bee Subspecies Breeding Zone Allocation was created between 1964 and 1979 based on the practices of private and commercial beekeepers, as well as researchers. It was officially accepted in 1979 [74]. This strategic initiative aimed to optimize the use of honey bee subspecies across Russia’s regions, with *A. m. mellifera* recommended for breeding in the Perm region. The plan sought to enhance bee sustainability and productivity by aligning breeds with regional climates and geography [65]. However, despite its advantages, it also faced significant opposition [75].

Although the Ministry of Agriculture currently oversees the plan, it is not a law and does not prohibit the introduction of other subspecies. As a result, beekeepers are responsible for honey bee subspecies on their farms, with hybridization posing challenges to beekeeping at the regional level.

This plan was not included in Federal Law No. 490-FZ “On Apiculture” (2020), which establishes the legal framework for beekeeping and bee conservation in Russia, defining apiculture as the breeding, keeping, and use of bees for pollination and product production (FAO, 2023, https://www.fao.org/faolex/results/details/en/c/LEX-FAOC200679/?utm accessed on 1 February 2025). While regional adaptations are allowed, the Perm region currently lacks specific legislation on apiculture, impeding local beekeeping development and regulation.

To address this and other communication gaps between researchers, individual and commercial beekeepers, and the government, over 900 registered nonprofit and commercial beekeeping organizations exist in Russia (personal communication). In the Perm region, the Beekeepers’ Association plays a key role in bridging the gap and advancing beekeeping and technology in the region. Beekeepers and members of the association attend open meetings of institutions such as the FSBSI “Federal Scientific Center of Beekeeping” (Ryazan region) and the Federal Scientific Institute of the North East (Kirov region). These discussions are incorporated into future research and breeding efforts, helping to develop new strategies for marketing beekeeping products both within Russia and internationally. However, as in all agricultural fields, there remains a need to strengthen communication channels between the government, scientists, and practitioners to address the interests of beekeepers, particularly those in remote regions.

## 6. Future Prospects for Conservation and Breeding in Russia

1. Selective breeding and reproduction—Selection of queen bees with relevant traits, their reproduction, and testing to ensure high-quality genetic traits for commercial and conservation purposes.
2. Controlled mating and genetic preservation—Establishment of mating stations to facilitate the controlled mating of virgin queens in isolation from other populations and breeds, preventing genetic dilution.
3. Conservation of *A. m. mellifera* habitats—Expansion of protected natural areas (PNA) and state-designated protection zones around breeding apiaries to preserve the gene pool and prevent genetic contamination from non-native honey bee populations.
4. Collaborative research and expertise exchange—Strengthening national and international cooperation with honey bee breeders to integrate advanced queen-rearing methods and enhance genetic selection programs.
5. Systematic queen distribution—Developing structured supply chains for *A. m. mellifera* queens to support conservation efforts, maintain genetic diversity, and promote sustainable beekeeping across Russia and internationally.
6. Communication and knowledge transfer—Establishing direct communication channels between researchers and beekeepers to facilitate the exchange of scientific knowledge, improve colony management, and enhance breeding outcomes.
7. Expansion of purebred colonies—Encouraging the increase in *A. m. mellifera* purebred colonies in private and commercial apiaries to safeguard genetic integrity and counteract uncontrolled hybridization with non-native bee populations.

## 7. Conclusions

Beekeeping in the Perm region provides key insights into managing honey bee populations under extreme climatic conditions. Targeted queen-rearing and colony management practices have helped preserve the genetic integrity of local *A. m. mellifera* populations, mitigating hybridization risks with non-native southern subspecies. Morphological, genetic, physiological, and ecological assessments confirm the adaptation of *A. m. mellifera* in the region, with northern (Krasnovishersky) and southern (Osinskaya and Uinskaya) Prikamskaya populations showing slight deviations in forewing length and cubital index while remaining within the breed standard (60–65%). Genetic and physiological analyses further highlight differences in overwintering metabolism and colony development. Despite conservation efforts, hybridization and limited commercial utilization of honey bee colonies underscore the need for improved breeding and management strategies.

Controlled breeding programs, adapted queen-rearing methods for cold climates, and drone population regulation have enhanced the maintenance of purebred colonies. These approaches are critical for preserving the cold-resistant traits of northern honey bee populations. Given ongoing climate change and increasing hybridization threats, the Perm region’s conservation strategies offer a valuable model for northern beekeeping in Europe, Canada, and the United States. Strengthening sustainable breeding practices and genetic protection measures is essential for ensuring the resilience of native bee populations and the stability of global pollination networks.

## Figures and Tables

**Table 1:** Morphometric characteristics of the northern (Krasnovisherskaya), and southern (Osinskaya, Uinskaya) Prikamskaya populations of *A. m. mellifera* L. honey bees in the Perm region (traits—mm, tarsal index—%).

Est.	Length of Proboscis (mm)	Length of Tergite 3 (mm)	Width of Tergite 3 (mm)	Length of Forewing (mm)	Width of Forewing (mm)	Cubital Index (%)	Tarsal Index (%)	Length of Sternite 4 (mm)
	Standard *A. m. mellifera* L.							
Min-max	6.0–6.4 *	−	−	9.3–9.6 *	2.9–3.3 *	60–65 *	52–58 *	−
Cv, %	5.9–6.4 **	2.4 **	5.0 **	−	−	−	54.0–55.5 *	3.0 **
	Northern (Krasnovisherskaya)							
M ± SEM	6.00 ± 0.007 *****	2.30 ± 0.002 *****	4.80 ± 0.005 *****	9.30 ± 0.008 *****	3.10 ± 0.004 *****	61.6 ± 0.34 *****	n.a	3.05 ± 0.003 ****
Cv, %	1.7	3.0	2.6	1.9	3.6	10.2	n.a	2.6
	*n* = 240	*n* = 240	*n* = 240	*n* = 240	*n* = 240	*n* = 240	n.a	*n* = 590
	Southern (Osinskaya, Uinskaya)							
M ± SEM	6.05 ± 0.008 ***	2.41 ± 0.004 ***	5.00 ± 0.008 ***	9.20 ± 0.008 *****	3.20 ± 0.004 *****	64.1 ± 0.65 ***	n.a	2.99 ± 0.002 ****
Cv, %	2.5	2.5	2.3	2.9	3.6	14.9	n.a	3.9
	*n* = 530	*n* = 950	*n* = 676	*n* = 870	*n* = 640	*n* = 530	n.a	*n* = 755

Note: * [64]; ** [65]; *** [66]; **** non-published data: Petukhov A. V.; ***** [67]; n.a = not available. M—mean, SEM—standard error of the mean, Cv—coefficient of variation. Cubital index was calculated by Alpatov [4].

## Data Availability

The original contributions presented in this study are included in the article. Further inquiries can be directed to the corresponding authors.
